# THDP17 Decreases Ammonia Production through Glutaminase Inhibition. A New Drug for Hepatic Encephalopathy Therapy

**DOI:** 10.1371/journal.pone.0109787

**Published:** 2014-10-17

**Authors:** M. Mar Díaz-Herrero, José A. del Campo, Pilar Carbonero-Aguilar, José M. Vega-Pérez, Fernando Iglesias-Guerra, Ignacio Periñán, Francisco J. Miñano, Juan Bautista, Manuel Romero-Gómez

**Affiliations:** 1 Departamento de Bioquímica y Biología Molecular, Facultad de Farmacia, Universidad de Sevilla, Sevilla, Spain; 2 Unidad de Gestión Clínica de Enfermedades Digestivas & Centro de Investigación Biomédica en Red de Enfermedades Hepáticas y Digestivas (CIBERehd), Hospital Universitario de Valme, Sevilla, Spain; 3 Departamento de Química Orgánica y Química Farmacéutica, Facultad de Farmacia, Universidad de Sevilla, Sevilla, Spain; 4 Unidad de Farmacología Experimental y Clínica (UFEC), Hospital Universitario de Valme, Universidad de Sevilla, Sevilla, Spain; Taipei Medical University, Taiwan

## Abstract

Ammonia production is implicated in the pathogenesis of hepatic encephalopathy (HE), being intestinal glutaminase activity the main source for ammonia. Management of ammonia formation can be effective in HE treatment by lowering intestinal ammonia production. The use of glutaminase inhibitors represents one way to achieve this goal. In this work, we have performed a search for specific inhibitors that could decrease glutaminase activity by screening two different groups of compounds: i) a group integrated by a diverse, highly pure small molecule compounds derived from thiourea ranging from 200 to 800 Daltons; and ii) a group integrated by commonly use compounds in the treatment of HE. Results shown that THDP-17 (10 µM), a thiourea derivate product, could inhibit the intestinal glutaminase activity (57.4±6.7%). Inhibitory effect was tissue dependent, ranging from 40±5.5% to 80±7.8% in an uncompetitive manner, showing V_max_ and K_m_ values of 384.62 µmol min^−1^, 13.62 mM with THDP-17 10 µM, respectively. This compound also decreased the glutaminase activity in Caco-2 cell cultures, showing a reduction of ammonia and glutamate production, compared to control cultures. Therefore, the THDP-17 compound could be a good candidate for HE management, by lowering ammonia production.

## Introduction

Ammonia plays a major role in the pathogenesis of hepatic encephalopathy (HE). Systemic hyperammonemia has been largely found in patients with cirrhosis and HE. Ammonia derived from the gut and kidneys must be detoxified in the liver and muscle. In cirrhotic patients with impaired capacity for detoxification of ammonia into urea, peripheral glutamine synthase (mainly in muscle tissue) serves as the main alternative for ammonia detoxification pathway. In the brain, hyperammonemia promotes astrocyte swelling and impairment of neurotransmission [1], [2]. In muscle tissue, it has been shown that glutaminase activity is increased in an animal model undergoing bile duct ligation and glutamine synthase has been shown to have an important role in detoxification of plasma ammonia in acute liver failure [3]. Moreover, the development of portosystemic shunting and alteration of blood flow is thought to affect circulating ammonia levels in cirrhosis [4]. These findings suggest that approaches to target glutaminase (by inhibition) may provide targets for ammonia detoxification as a valid therapeutic strategy for HE.

Glutamine deamidation by intestinal GA has been proposed as one of the main sources of ammonia production in patients with cirrhosis [5]. However, hyperammonemia found after portacaval shunt in rats is similar in both germ-free and non-germ-free animals [6], [7] supporting the hypothesis that hyperammonemia and encephalopathy can be developed without the participation of bacteria [8].

Another supporting evidence is that the greatest hyperammonemia has been found in portal-drained viscera and derived mainly from glutamine deamidation in cirrhotic patients by phosphate-activated glutaminase (PAG) [9], [10]. Glutamine synthesis is an effective mechanism to reduce the levels of ammonia; however, much of the newly synthesized glutamine is subsequently metabolized in mitochondria by phosphate-activated glutaminase, yielding glutamate and ammonia. In this manner, glutamine (the Trojan horse) is transported in excess from the cytoplasm to mitochondria serving as a carrier of ammonia [11].

PAG catalyzes the deamidation of glutamine to yield glutamate and ammonia. There are two genes that encoding two main isoforms of PAG: the kidney-type PAG (K-PAG) and the hepatic-type PAG (L-PAG). The K-PAG is the ubiquitous form. It can be found in kidney, brain, stomach, pancreas, muscle, or villus enterocyte; while L-PAG was thought to be restricted to the liver but in the last years it has also been found in nuclei of mammalian brain [12], and granule in human neutrophils [13]. The presence of LGA and/or KGA, together with GAC (Glutaminase C) form has also been reported in different tumour cells [14]–[16].

In healthy people, the higher PAG activity along the gastrointestinal tract has been found in the small intestine, being higher than 80% of the activity [17]. Lower but still substantial activity was found in the large intestine, approximately the 15%. In rats, the distribution of the PAG activity was found to be similar to humans [18]. Duodenal PAG activity, measured in mucosal biopsies from the first portion of the small intestine, has been found to be nearly four-times higher in cirrhotic patients than in healthy controls [10].

As the main source of ammonia in cirrhotic patients derives from portal-drained viscera owing to glutamine deamidation, PAG activity in the intestine seems to be responsible, largely, for systemic hyperammonemia production in these patients [5]. Besides, in portacaval shunted rats, a model for minimal HE [19], PAG activity has been found to be increased in duodenum and ileum compared to sham-operated rats [20]. All these data suggest that PAG is implicated in the production of systemic hyperammonemia from the intestine and could play a major role in the pathogenesis of HE. Therefore, a decrease of ammonia production in the small intestine by PAG inhibition may contribute to lower ammonia concentration in the systemic circulation. The aim of this work was the search for glutaminase inhibitors in order to find new ammonia-lowering drugs that could be used in the management of HE.

## Materials and Methods

All chemicals used in this work were from high analytical grade and were obtained mainly from Sigma-Aldrich.

### Compounds

Two groups of compounds were used:

The first group was integrated by a collection of diverse, highly pure, small molecule compounds derived from thiourea ranging from 200 to 800 Daltons. These compounds were named as THDP-xx, being xx an assigned number to each product. Compounds were received as powder and then dissolved in dimethyl sulfoxide (DMSO) to constitute a 10 mM stock concentration and stored at −20°C. Maximum DMSO concentration in the reaction mixture was 0.5% (vol/vol) to avoid toxic effects. Most of the compounds in the library can be dissolved at 50 µg/ml. For those compounds that did not dissolve (∼5%), concentration was decreased to 10–25 µg/ml. All compounds were synthesized at the Departamento de Química Orgánica y Farmacéutica of Universidad de Sevilla (Spain) [21]–[23] as the result for a new simple thiourea derivatives library with potential use as enzymatic inhibitors.

The second group was integrated by compounds used or tested for the treatment of HE: metformine, metronidazole, neomicine, rifaximin, lactulose and lactitol (all purchased from Sigma-Aldrich).

### In vitro inhibition screening

PAG inhibition analysis by different thiourea derivatives (THDP) was assayed *in vitro*, using the method of Heini for glutaminase activity determination [24], adapted to a microtiter procedure. This assay is based on the use of o-phthaldialdehyde/mercaptoethanol as a reagent for estimating ammonia released from glutamine. Briefly, 25 µl samples were added to 35 µl of a mixture of 150 mM potassium phosphate, 171 mM glutamine, 1 mM ammonium chloride, pH 8.0 and then incubated at 37°C for 45 min. The reaction was stopped by adding 10 µl 7% trichloroacetic acid, kept on ice for 15 min and centrifuged 5 min at 1000 × g. Aliquots of 5 µl/well were then mixed with 150 µl o-phthalaldehyde/mercaptoethanol reagent (14.4 ml 200 mM potassium phosphate, pH 7.4, 0.8 ml 72 mM mercaptoethanol in ethanol and 0.8 ml 186 mM o-phthaldialdehyde in ethanol). The samples were kept at room temperature in the dark and absorbance measured at 410 nm with a UV-Visible spectrophotometer (BioRad) after 45 min.

### Kinetic assays

K-PAG activity was assayed, in presence and absence of the inhibitor; using isolated mitochondria from rat intestine and kidney, solubilized with 1% Triton X-100 as a source of glutaminase [25]. Enzymatic activity was measured using a two-step assay as described by Lund [26]. Protein concentration was determined following Bradford method using serum albumin as standard [27].

### Cell culture

Caco2 cells were purchased from Sigma-Aldrich (St. Louis, MO, USA) and grown with DMEM (Dulbecco’s Modified Eagle Medium) medium supplemented with 2 mM L-Glutamine, 15% FBS, 1X antinbiotic/antimicotic solution, 1X non-essential amino acids (PAA Laboratories GmbH, Linz, Germany). Cells were incubated at 37°C and 5% CO_2_.

**Table 1:** THDP-17 was administered orally after 12 h fasted mice.

Group	N	Vehicle(DMSO)	THDP-17(mg/Kg)	Deaths before		Sacrificed 24 h
				6 h	12 h	
I	3	+	0	1	1	1
II	3	+	50	1	1	1
III	3	+	300	1	1	1
IV	3	+	2000	2	1	–

Animals were divided in 4 groups and deaths after treatment were recorded.

### Cytotoxicity assay

Cytotoxicity was evaluated in Caco-2 cells after 24 h-exposure to selected inhibitors using neutral red (NR) assay (Zhang et al. 1990) [28]. Regression analysis of dose-response curves and calculation of IC_50_ were done according to standard procedure using appropriated software.

### Inhibition assay in cell culture

Inhibition assay was carried out by measuring the concentration of glutamate produced from glutamine by Caco-2 cells using HPLC, in presence and absence of the selected inhibitor (THDP-17) at different concentrations (0, 5, 20 and 100 µM) and time points (24 and 48 h). 6-Diazo-5-oxo-L-norleucine (DON) (0–100 µM) was used as positive control. The presence of the THDP-17 in the cells was checked by HPLC-MS.

### Lethal Dose 50

Lethal dose was determined by oral administration of the compounds to C57BL/6 mice. Thirty mice were supplied by the Centre of Production and Experimental animal of Espartinas, University of Seville, Spain. The animals were housed ten per cage, and maintained at constant room temperature (22°C ±2°C), with artificial light-dark cycle of 12 h and a relative humidity of 65%−70%, and free access to food and water during 2 weeks for acclimation. All animal procedures were performed with approval from the Sevilla University Animal Ethics Committees, under the guidelines of the directive 86/609/CEE of the European Union. Two assays in different conditions were performed. In the first assay fasted mice were divided in four groups of three animals and were administered 0, 50, 300 or 2000 mg THDP-17/kg, respectively. They were sacrificed 24 h latter and a macroscopic analysis of the tissues (lungs, heart, liver, stomach and intestine) was performed. In the second assay the mice were feed ad libitum, separated into three groups of four animals per group and administered them 0, 5 or 300 mg THDP-17/kg, respectively. In this case, mice were sacrificed 24 or 72 h latter and the same macroscopic analysis was performed.

### Statistical analysis

The results were expressed as means and standard deviations (or standard errors) of three independent experiments. The results were analyzed with the Student t test. P<0.001, P<0.01, and P<0.05 were considered statistically significant.

## Results and Discussion

Ammonia-lowering drugs have received great interest in the management of HE in the last few years. In order to develop adequate and selective inhibitors of intestinal PAG activity, we performed a screening of three different groups of compounds: i) a group integrated by a collection of diverse, highly pure, rationally selected small molecule compounds derived from thiourea ranging from 200 to 800 Daltons; ii) a group integrated by products showing some structural analogy with the compound THDP-17, and iii) a group integrated by compounds usually used in the treatment of HE.

### i) Thiourea derived products

The first group analysed in this work is integrated by a small molecule compounds library derived from thiourea [21]. We found that some products derived from thiourea were able to reduce ammonia production *in vitro* from glutamine by an enriched preparation of glutaminase obtained from both kidney and intestine mitochondria (data not shown). For the screening, we have used the Heini’s method to assay the glutaminase activity. Six synthetic inhibitors derived from thiourea were selected and extensively characterized. These products (THDP-17, THDP-33, THDP-39, THDP-41, THDP-42, and THDP-43) could inhibit K-PAG activity and were tested for toxicity and grade of inhibition on a cell culture based method, using the neutral red (NR) assay.

#### - Effects of selected inhibitors on cytotoxicity

Acute toxicity test in animals is typically the first step in the evaluation of the effects of a chemical compound, and its primary purpose is to provide information on potential health hazards that may result from a short-term exposure. Recent studies suggest that *in vitro* methods might be helpful in predicting acute toxicity and estimating toxic chemical concentrations in vivo [29]. In the present work, cytotoxicity of a 24 h-exposure to selected inhibitors was evaluated in Caco-2 cells using the MTT method developed by Mosmann [30] and modified by Denizot and Lang [31]. Based on cell viability the inhibitors THDP-41, THDP-42 and THDP-43 were rejected due to their high toxicity and the remaining compounds (THDP-17, THDP-33 and THDP-39) were selected for further inhibition studies.

#### - In vitro inhibition analysis

**Figure 1:**
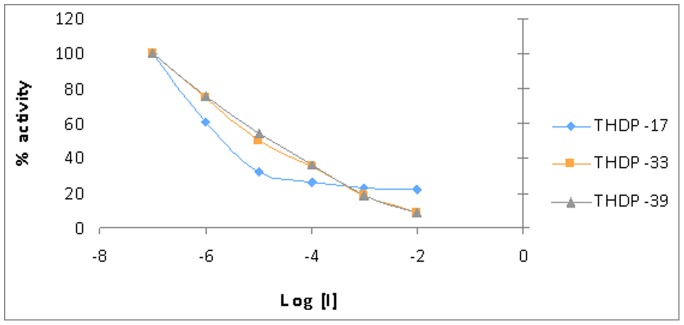
Inhibitory effect on glutaminase activity of several compounds tested in this study. Data are shown in log scale (inhibitor concentration), being 100% activity measured without inhibitor. There was not significant differences among compounds tested (Student t test; p>0.05). THDP-17 was chosen on toxic effect base.

To evaluate the performance of the PAG-inhibitors THDP-17, THDP-33 and THDP-39 we used swine K-PAG (from kidney) in an *in vitro* optimised kinetic assay with and without the addition of the inhibitors. First, increasing concentration of inhibitors (ranging from 1 µM to 10 mM) were added to standard and optimized enzymatic assay conditions with 99 mmol/l glutamine, 150 mmol/l phosphate and 100 µg K-PAG (Figure 1). THDP-33 and THDP-39 were able to inhibit PAG completely at 10 mM. IC_50_ values were 19.1±1.3 µM for THPD-33, 31.6±2.7 µM for THDP-39 and 3.9±0.1 µM for THDP-17, indicating that THPD-33 and THPD-39 were less powerful inhibitors than THDP-17. THDP-33 and THDP-39 were able to completely inhibit PAG activity at low milimolar level (Figure 1) which can be harmful for the enterocyte, being discarded for future analysis.

**Figure 2:**
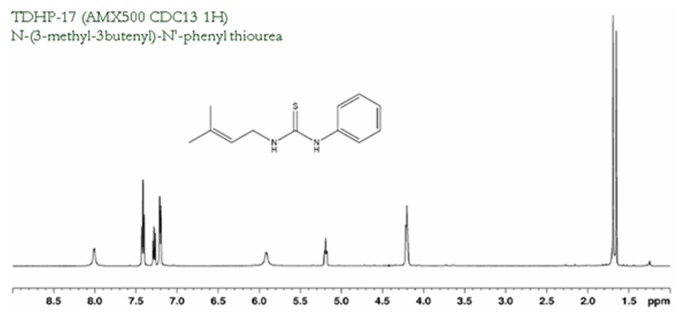
Molecular structure of N-(3-methyl 3-butenyl)-N′ phenyl thiourea (THDP17).

THDP-17, N-(3-methyl-3-butenyl)-N′-phenylthiourea (Figure 2), was selected because it did not show cytotoxicity in cell culture, and because it inhibits PAG activity partially at low milimolar concentrations. In fact, there is a plateau (the enzyme activity is no longer affected by increasing the inhibitor concentration). This finding is highly relevant, since partial inhibition of PAG could be beneficial for the reduction of ammonia and the excess of glutamate production in the management of HE, without affecting the viability of the enterocyte.

**Figure 3:**
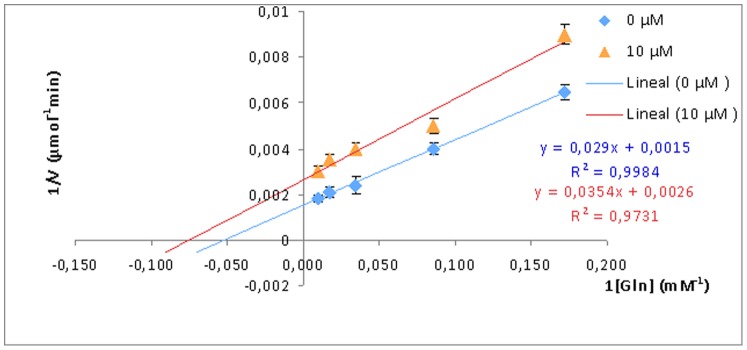
THDP17 inhibition kinetics. A lineal double reciprocal plot (1/v against 1/[Gln]) in absence (0) and presence (10 µM) of the inhibitor is shown. V_max_ and K_M_ values for PAG were 0.67 mmol min^−1^, and 19.33 mM, respectively. In the presence of THDP-17 both parameters were modified: V_max-ap_ = 0.38 mmol min^−1^, and K_M-ap_ = 13.62 mM, indicating an uncompetitive inhibition. All experiments were performed in triplicates; p>0.05.

PAG kinetic parameters were analyzed in absence and presence of THDP-17, at different concentrations (1 and 10 µM), under optimal analytical conditions, at different glutamine concentration (0 to 200 mM). A lineal double reciprocal plot (1/v against 1/[Gln]) in absence and presence of the inhibitor (Figure 3) shows that the V_max_ and K_M_ values for PAG were 0.67 mmol min^-1^, and 19.33 mM, respectively, indicating that in presence of the inhibitor THDP-17 both parameters changes: V_max-ap_ = 0.38 mmol min^−1^, and K_M-ap_ = 13.62 mM). Data show an uncompetitive inhibition model indicating that THDP-17 binds PAG at a site created after enzyme-substrate complex formation. We propose that THDP-17 is a not-pure-uncompetitive inhibitor because the K_i_ value obtained from V_max-ap_ and K_M-ap_ (23.2 mM and 40.5 mM, respectively) is different.

#### - *in vivo* inhibition

**Figure 4:**
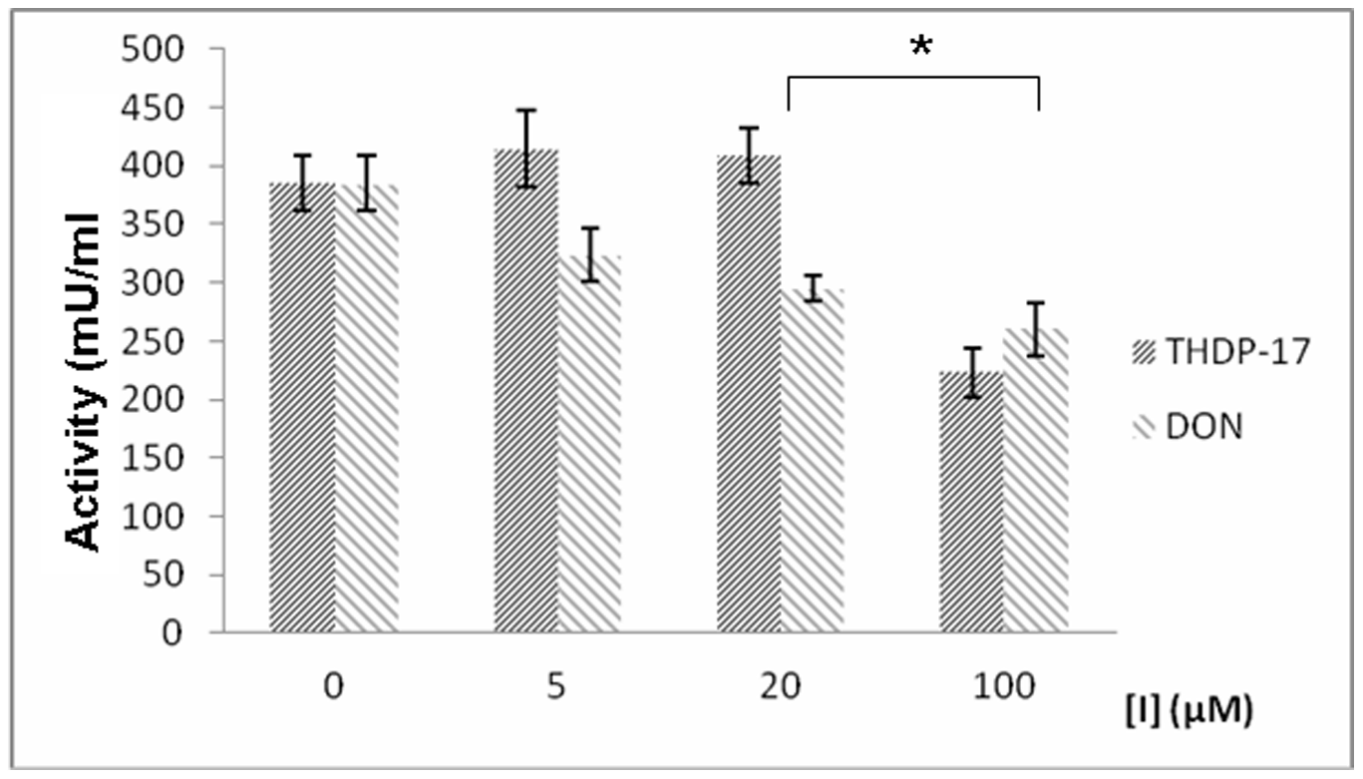
*In vivo* inhibitory effect on glutaminase activity at different concentrations of THDP17 and DON (6-Diazo-5-oxo-L-norleucine) was tested in Caco-2 cell cultures. PAG inhibition by THDP-17 in cell cultures is effective at concentration higher than 5 µM, showing an inhibition of 18±2.1% and 46±3.4% at 20 and 100 µM, respectively (* p<0.05).

Since *in vitro* inhibition by THDP-17 does not guarantee that the inhibitor also works *in vivo*, we evaluated the production of ammonia and glutamate from glutamine by enterocyte cells (Caco-2) in the presence and absence of THDP-17 at different concentrations (0, 5, 20 and 100 µM). Glutamate concentration was determined by HLPC in the supernatants and the presence of THDP-17 in the cells was analyzed by HPLC-MS. Results are shown on Figure 4. These data indicate that PAG inhibition by THDP-17 in cell cultures is effective at a concentration higher than 5 µM, showing an inhibition of 18±2.1% and 46±3.4% at 20 and 100 µM, respectively. This fact can be explained assuming that the inhibitor can diffuse or transported through the plasma and the outer mitochondrial membranes, reaching the PAG located at the inner mitochondrial membrane, interfering its function and lowering its activity. Since there is no direct evidence that this transport is taking place, we have studied the evolution of the concentration of THDP in the medium along the cultivation period, showing that 56% and 72% of the inhibitor is diffused or transported into the cells at 20 and 100 µM, respectively.

#### *-* Acute Toxicity of THDP17

Although THDP-17 did not show any cytotoxic effect in cell cultures, we have studied the effect of this compound ***in vivo*** using an animal model (C57BL mice) since thiourea-derived compounds can be toxic ***in vivo*** [32]. Two different assays were carried out: first, THDP-17 was administered orally after 12 h fasted. In this case deaths were observed at 6, 12 and 24 h in all groups (Table-1), including control group (without THDP-17) where only vehicle (DMSO) was administered. However, lethality of DMSO-treated mice did not differ significantly in its lethality as compared to the groups treated with THDP-17. The undiluted compound has low systemic toxicity but a marked local necrotizing and inflammatory effect when it is administered orally. Animals showed gastrointestinal ulcers and duodenum bleeding (Figure S1). There were few fluctuations in histopathological values but no changes appear to be drug-related or of significance. No essential or histopathological damages were seen in mice treated with THDP-17 including lungs, heart or liver when compared to DMSO alone. We interpreted these results as DMSO and/or THDP-17 was toxic for the animals in fasted state. Therefore, we repeat the assay using feed animals. THDP-17 and/or DMSO were then administered orally to animals after free access to food and water overnight, and mice were sacrificed at 24 h and 72 h randomly. In this case, no deaths were observed during the experimental period (Table 2); probably due to protective effect of food. Macroscopic organs analysis from animals sacrificed at 24 and 72 h showed gastrointestinal ulcers and duodenum bleeding, although less pronounced than in the case of the first group (fasted group).

**Table 2:** THDP-17 and/or DMSO were then administered orally to animals after free access to food and water overnight, and mice were sacrificed at 24 h and 72 h randomly.

Group	N	Vehicle(DMSO)	THDP-17(mg/Kg)	Deaths before			Sacrificed	
				6 h	12 h	24 h	24	72
I	4	+	0	0	0	0	2	1
II	4	+	50	0	0	0	2	2
III	4	+	300	1*	0	0	1	2

*Animal without water.

These results demonstrate that THDP-17 is no toxic *in vivo* and suggest that DMSO enhanced the toxicity of THDP in mice. Therefore, this compound is currently used as a primary group for the synthesis amphiphilic derivatives.

### ii) Products currently used in HE therapy

A second group of compounds integrated by drugs habitually used in the treatment of HE was also tested for glutaminase inhibition: metformin, metronidazole, neomicine, rifaximin, lactulose and lactitol. PAG activity inhibition was analyzed using 10 mM concentration. PAG activity *in vitro* was no affected by neomycin, lactitol and lactulose, rather, an increased activity was observed.

**Figure 5:**
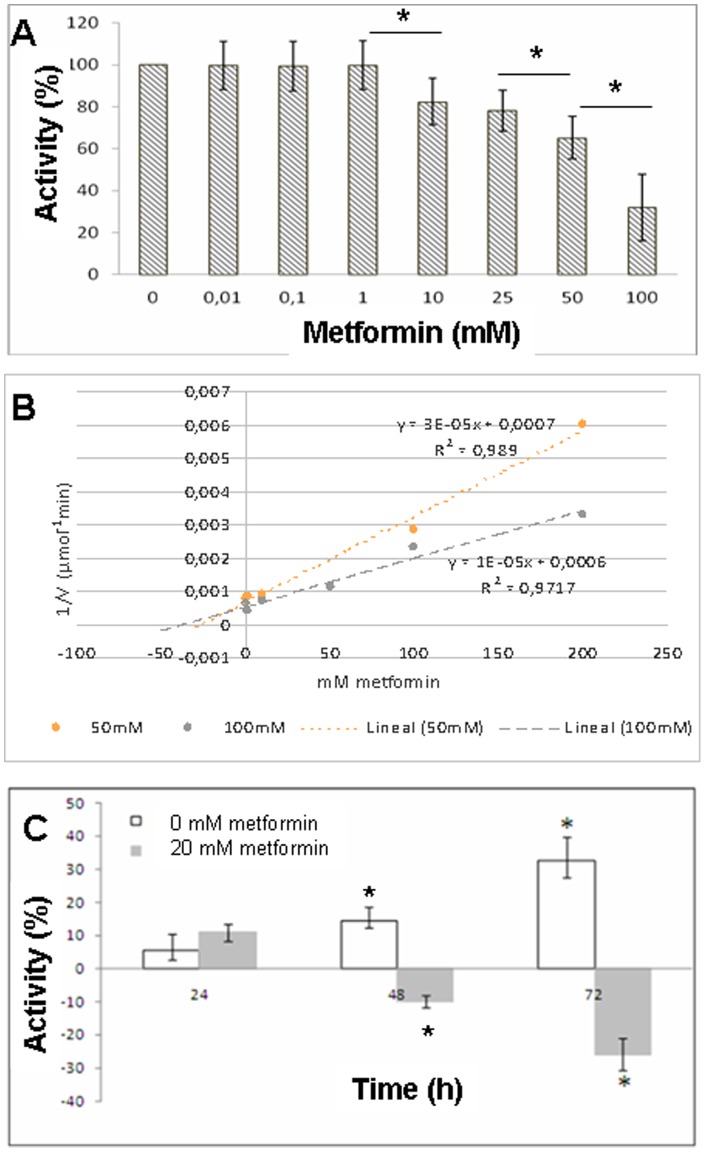
Metformin inhibits glutaminase activity in a dose dependent manner: from 17.5% at 10 mM up to 68% at 100 mM (A) (* p<0.05). This effect, analyzed by Dixon plot, showed competitive inhibition kinetics, with a K_i_ of 14.28 mM (B). In Caco2 cells, metformin 20 mM showed 24% inhibition of glutaminase activity at 72 hours compared to control cultures (p<0.05) (glutamate production was decreased from 26.85±0.74 µM to 19.9±2.05 µM; p<0.05) (C).

Since metformin seems to be protective against hepatic encephalopathy in diabetic cirrhotic patients [33], we decided to analyze in vitro PAG inhibition by metformin. Results in Figure-5 show that PAG was inhibited in a dose dependent manner: from 17.5% at 10 mM up to 68% at 100 mM. This effect, analyzed by Dixon plot, showed competitive inhibition kinetics, with a K_i_ of 14.28 mM (Figure 5B). In Caco2 cells, metformin 20 mM showed 24% inhibition of glutaminase activity at 72 hours compared to control cultures (p<0.05) (glutamate production was decreased from 26.85±0.74 µM to 19.9±2.05 µM; p<0.05) (Figure-5C). Higher metformin concentrations (50, 100 and 200 mM) did not yield higher inhibition rates. These results demonstrated that metformin could serve as a new therapeutic option for HE management, although THDP17 was able to inhibit glutaminase (46±3.4%) in a higher extent.

### Conclusions

The major findings in this work are: i) although THDP-17 was reported first in 1960, this is the first time that its use as glutaminase activity inhibitor is described. THDP17 shows a partial uncompetitive inhibition *in vitro* and a significant reduction of ammonia and glutamate production *in vivo*. Therefore, THDP-17 could be a good candidate for HE management by lowering ammonia production. ii) Moreover, we have shown that metformin, an insulin sensitizer drug used for diabetes therapy, could also inhibit PAG activity, resulting in new therapeutic option for HE by reducing splenic ammonia production.

## Supporting Information

Figure S1 **Fasted C57BL/6 mice were treated with either DMSO (vehicle) or THDP-17.** Both groups showed acute toxicity effects. A- Internal general appearance, showing gastrointestinal ulcers and bleeding in the duodenum; B- Bowels; C- Stomach.
(TIF)

## References

[1] Zemtsova I, Görg B, Keitel V, Bidmon HJ, Schrör K, et al (2011) Microglia activation in hepatic encephalopathy in rats and humans. Hepatology 54: 204–15. 21452284 10.1002/hep.24326

[2] Llansola M, Montoliu C, Cauli O, Hernández-Rabaza V, Agustí A, et al (2013) Chronic hyperammonemia, glutamatergic neurotransmission and neurological alterations. Metab Brain Dis. 28: 151–154. 10.1007/s11011-012-9337-3 23010935

[3] Jover-Cobos M, Noiret L, Lee K, Sharma V, Habtesion A, et al (2014) Ornithine phenylacetate targets alterations in the expression and activity of glutamine synthase and glutaminase to reduce ammonia levels in bile duct ligated rats. J Hepatol. 60: 545–53. 10.1016/j.jhep.2013.10.012 24512823

[4] Dam G, Keiding S, Munk OL, Ott P, Vilstrup H, et al (2013) Hepatic encephalopathy is associated with decreased cerebral oxygen metabolism and blood flow, not increased ammonia uptake. Hepatology 57: 258–265. 22886493 10.1002/hep.25995

[5] Romero-Gómez M, Bautista JD, Grande L, Ramos Guerrero RM, Sanchez Munoz D (2004) New concepts in the physiopathology of hepatic encephalopathy and therapeutic prospects. Gastroenterol Hepatol. 27: 40–8. 15195533

[6] Nance FC, Kline DG (1971) Eck’s fistula encephalopathy in germ-free dogs. Ann Surg. 174: 856–61. 10.1097/00000658-197111000-00018 PMC1397554 5113466

[7] Warren KS, Newton WL (1959) Portal and peripheral blood ammonia concentrations in germ-free and conventional guinea pigs. Am J Pysiol. 197: 717–20. 10.1152/ajplegacy.1959.197.3.717 13842938

[8] Weber FJL, Veach GL (1979) The importance of the small intestine in gut ammonium production in the fasting dog. Gastroenterology 77: 235–40. 447037

[9] Olde-Damink SW, Jalan R, Redhead DN., Hayes PC, Deutz NE, et al.. (2002) Inter-organ ammonia and amino acid metabolismo in metabolically stable patients with cirrhosis and a TIPSS. Hepatology 36; 1163–71. 10.1053/jhep.2002.36497 12395326

[10] Romero-Gómez M, Ramos-Guerrero R, Grande L, de Terán LC, Corpas R, et al (2004) Intestinal glutaminase activity is increased in liver cirrhosis and correlates with minimal hepatic encephalopathy. J Hepatol 41: 49–54. 15246207 10.1016/j.jhep.2004.03.021

[11] Albrecht J, Norenberg MD (2006) Glutamine: a Trojan horse in ammonia neurotoxicity. Hepatology 44: 788–94. 17006913 10.1002/hep.21357

[12] Olalla L, Gutiérrez A, Campos JA, Khan ZU, Segura JA, et al (2002) Nuclear localization of L-type glutaminase in mammalian brain. J Biol Chem 277: 28939–38944. 10.1074/jbc.C200373200 12163477

[13] Castell L, Vance C, Abbott R, Márquez J, Eggleton P (2004) Granule localization of glutaminase in human neutrophils and the consequence of glutamine utilization for neutrophil activity. J Biol Chem 279: 13305–10. 14722097 10.1074/jbc.M309520200

[14] Pérez-Gómez C, Campos-Sandoval JA, Alonso FJ, Segura JA, Manzanares E, et al (2005) Co-expression of glutaminase K and L isoenzymes in human tumour cells. Biochem J 385: 535–542. 10.1042/BJ20040996 PMC1134872 15496140

[15] Roberg BA, Torgner IA, Kvamme E (2010) Kinetics of a novel isoform of phosphate activated glutaminasa (PAG) in SH.SY5Y neuroblastoma cells. Neurochem Res 35: 875–80. 19894115 10.1007/s11064-009-0077-7

[16] Romero-Gómez M, Jover M, Del Campo JA, Royo JL, Hoyas E, et al (2010) Variations in the promoter region of the glutaminase gene and the development of hepatic encephalopathy in patients with cirrhosis: a cohort study. Ann Intern Med.153: 281–288. 20820037 10.7326/0003-4819-153-5-201009070-00002

[17] James LA, Lunn PG, Middleton S, Elia M (1998) Distribution of glutaminase and glutamine synthase activities in the human gastrointestinal tract. Clin Sci. 94: 313–19. 10.1042/cs0940313 9616266

[18] James LA, Lunn PG, Elia M (1988) Glutamine metabolism in the gastrointestinal tract of the rat assessed by the relative activity of glutaminase (EC 3.5.1.2) and glutamine synthetase (EC 6.3.1.2). Br J Nutrition. 79: 365–72. 10.1079/bjn19980061 9624228

[19] Butterwort RF, Norenberg MD, Felipo V, Ferenci P, Albrecht J, et al (2009) Experimental models of hepatic encephalopathy: ISHEN guidelines. Liver Int 29 783–788. 19638106 10.1111/j.1478-3231.2009.02034.x

[20] Hawkins RA, Jessy J, Mans AM, Chedid A, DeJoseph MR (1994) Neomycin reduces the intestinal production of ammonia from glutamine. Adv Exp Med Biol. 368: 125–34. 10.1007/978-1-4615-1989-8_13 7741004

[21] Vega-Pérez JM, Periñán I, Argandoña M, Vega-Holm M, Palo-Nieto C, et al (2012) Isoprenyl-thiourea and urea derivatives as new farnesyl diphosphate analogues: synthesis and in vitro antimicrobial and cytotoxic activities. Eur J Med Chem. 58: 591–612. 10.1016/j.ejmech.2012.10.042 23174318

[22] Iliceto A, Fava A, Mazzucato U, Radici P (1960) Allyl and benzhydryl thiocyanates and isothiocyanates; thiocyanate-isothiocyanate equilibrium. Gazzetta Chimica Italiana 90: 919–40.

[23] Furdik M, Sutoris V (1961) Synergists of pyrethrum. VI. Synthesis of endo-cis-N-substituted 7-diphenylmethylenebicyclo[1.2.2]hept-5-ene-2,-3-dicarboximides. Chemicke Zvesti 15: 173–80.

[24] Heini HG, Gebhardt R, Brecht A, Mecke D (1987) Purification and characterization of rat liver glutaminasa. Eur J Biochem 162: 541–546. 3830157 10.1111/j.1432-1033.1987.tb10673.x

[25] Shapiro RA, Haser WG, Curthoys NP (1985) The orientation of phosphate-dependent glutaminasa on the inner membrane of rat renal mitochondria. Arch Biochem Bioph 243: 1–7. 10.1016/0003-9861(85)90767-2 2998280

[26] Lund P (1988) L-glutamine and L-glutamate: UV-method with glutaminasa and glutamate dehydrogenase, in: H.U. Bermeyer (Eds.), Methods of enzymatic analysis, Weinheim, 57–363.

[27] Bradford MM (1976) Rapid and sensitive method for the quantitation of microgram quantities of protein utilizing the principle of protein-dye binding. Anal Biochem 72: 248–254. 942051 10.1016/0003-2697(76)90527-3

[28] Zhang SZ, Lipsky MM, Trump BF, Hsu IC (1990) Neutral red (NR) assay for cell viability and xenobiotic-induced cytotoxicity in primary cultures of human and rat hepatocytes. Cell Biol Toxicol. 6: 219–34. 10.1007/BF00249595 2113829

[29] Spielmann H, Genschow E, Leibsch M, Halle W (1999) Determination of the starting dose for acute oral toxicity (LD50) testing in the up and down procedure (UDP) from cytotoxicity data. Alternatives to Laboratory Animals 27: 957–966. 25490464 10.1177/026119299902700609

[30] Mossman T (1983) Rapid colorimetric assay for cellular growth and survival: application to proliferation and cytotoxicity assay. J. Immunol. Methods 65: 55–63. 10.1016/0022-1759(83)90303-4 6606682

[31] Denizot F, Lang R (1986) Rapid colorimetric assay for cellular growth and survival. Modifications to the tetrazolium dye procedure giving improved sensitivity and reliability. J. Immunol. Methods 89: 271–277. 10.1016/0022-1759(86)90368-6 3486233

[32] Smith PB, Crespi C (2002) Thiourea toxicity in mouse C3H/10T12 cells expressing human flavin-dependent monooxigenase 3. Biochemical Pharmacology 63: 1941–1948. 12093470 10.1016/s0006-2952(02)00978-4

[33] Ampuero J, Ranchal I, Nuñez D, Díaz-Herrero MM, Maraver M, et al (2012) Metformin Inhibits Glutaminase Activity and Protects against Hepatic Encephalopathy. PLoS One 7: e49279. 23166628 10.1371/journal.pone.0049279 PMC3499552

