# Supplementary figures and images for: THDP17 Decreases Ammonia Production through Glutaminase Inhibition. A New Drug for Hepatic Encephalopathy Therapy
Source: PLoS One. 2014 Oct 17;9(10):e109787. doi: 10.1371/journal.pone.0109787 (PMC4201470; doi:10.1371/journal.pone.0109787)

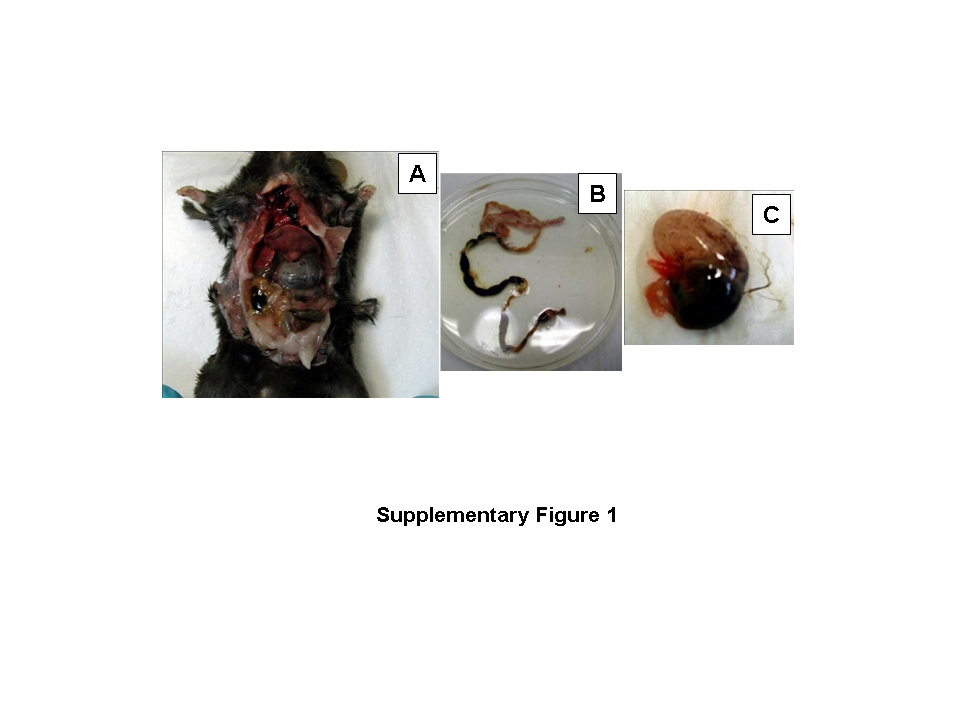

Supplement: Figure S1 — Fasted C57BL/6 mice were treated with either DMSO (vehicle) or THDP-17. Both groups showed acute toxicity effects. A- Internal general appearance, showing gastrointestinal ulcers and bleeding in the duodenum; B- Bowels; C- Stomach. (TIF) [file pone.0109787.s001.tif]
